# KDTMD: Knowledge distillation for transportation mode detection based on KAN

**DOI:** 10.1371/journal.pone.0324752

**Published:** 2025-06-02

**Authors:** Rui Li, Xueyi Song, Yongliang Xie

**Affiliations:** 1 Zhejiang Technical Institute of Economics, Hangzhou, Zhejiang, China; 2 China Construction Civil Engineering Co. Ltd., Beijing, China; Kafkas University: Kafkas Universitesi, TÜRKIYE

## Abstract

With the progress in sensor technology and the spread of mobile devices, transportation mode detection (TMD) is gaining importance for health and urban traffic improvements. As mobile devices become more lightweight, they require more efficient, low-power models to handle limited resources effectively. Despite extensive research on TMD, challenges remain in capturing non-stationary temporal dynamics and nonlinear fitting capabilities. Additionally, many existing models exhibit high space complexity, making lightweight deployment on devices with limited computing and memory resources difficult. To address these issues, we propose a novel deep TMD model based on discrete wavelet transform (DWT) and knowledge distillation (KD), called KDTMD. This model consists of two main modules, i.e., DWT and KD. For the DWT module, since non-stationary time variations and event distribution shifts complicate sensor time series analysis, we use the DWT modules to disentangle the sensor time series into two parts: a low-frequency part that indicates the trend and a high-frequency part that captures events. The separated trend data is less influenced by event distribution shifts, effectively mitigating the impact of non-stationary time variations. For the KD module, it includes the teacher model and student model. Specifically, for teacher model, to address the nonlinearities and interpretability, we incorporate T-KAN, which is composed of multiple layers of linear KAN that employ learnable B-spline functions to achieve a richer feature representation with fewer parameters. For student model, we develop the S-CNN, which is trained efficiently by T-KAN through KD. The KDTMD model achieves 97.27% accuracy and 97.29% F1-Score on the SHL dataset, and 96.56% accuracy and 96.72% F1-Score on the HTC dataset. Additionally, the parameters of the KDTMD model are only about 10% of the smallest baseline.

## Introduction

In contemporary society, smartphones, smartwatches, and other wearable devices, equipped with advanced sensors for efficient user data collection have become widespread. This capability has significantly advanced the development of Human Activity Recognition (HAR) technology [1]. A key application of HAR is Transportation Mode Detection (TMD), which identifies movement patterns through sensor data analysis. By analyzing movement patterns in real-time, TMD technology enhances traffic prediction [2], logistics route optimization [3], carbon footprint estimation [4], and other intelligent services. TMD technology has advanced from GPS-based approaches [5–8] to multi-sensor integration [9–12], and now utilizes deep learning and AI [13–16] for improved efficiency and accuracy.

In this paper, we aim to develop a lightweight model for TMD that can be directly deployed on mobile devices such as smartphones. The model is designed to accurately and swiftly determine which of the following eight specific transportation modes the user is in: being stationary, walking, running, cycling, driving, taking a bus, taking a train, and traveling by subway. *It is challenging to achieve this goal, as several key challenges remain to be addressed:*

**Fig 1:**
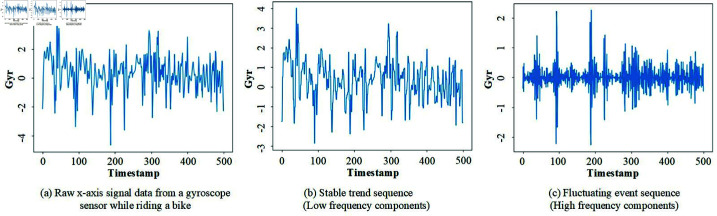
Raw x-axis signal data from a gyroscope sensor while riding a bike, showing stable trend and fluctuating event sequences (sampling frequency: 100 Hz, duration: 5s).

- **Non-stationary temporal dynamics.** In the complex time series and dynamic spatial correlation issues of TMD, understanding and capturing the non-stationary temporal changes and spatial dynamics are highly challenging for TMD. This is because the data we collect from sensors are usually entangled by a stable trend sequence and a fluctuating event sequence (as shown in Fig 1, the raw x-axis signal data collected from the gyroscope sensor of a smartphone while riding a bike includes both a stable trend sequence and a fluctuating event sequence, with a sampling frequency of 100 Hz over a duration of 5s.), where the fluctuating events frequently undergo distribution shifts. Common solutions for TMD generally feed sensor time series directly into the network. Those methods that did not distinguish between these two sequences struggled to avoid distribution shifts, making it challenging to make reasonable predictions.
- **Non-linear fitting capability.** Transportation data often exhibit complex nonlinear relationships, necessitating models that can capture this complexity. Traditional methods typically have notable shortcomings when it comes to fitting nonlinear functions. These shortcomings include a large number of parameters and poor interpretability. Additionally, these methods often struggle with high-dimensional data and may not be very effective in TMD problems due to their limited expressive capabilities [16].
- **Space complexity for lightweight application.** The remarkable success of deep learning largely stems from its ability to handle vast amounts of data and manage complex models with high computational demands. However, for TMD, which requires processing large volumes of sensor data, deploying these complex traditional deep learning models on resource-constrained devices such as mobile phones and embedded systems poses significant challenges due to their high computational demands and substantial storage needs. Traditional deep learning models often struggle to reduce this complexity while maintaining accuracy, especially when dealing with large-scale datasets [17].

To address the aforementioned challenges, this paper proposes a novel model based on Discrete Wavelet Transform (DWT) and Knowledge Distillation (KD), referred to as the KDTMD model for TMD. In our model, to capture the complex temporal relationships from short-term fluctuations to long-term trends, we first apply DWT to disentangle the sensor time series into a stable trend sequence and a fluctuating event sequence. To reduce space complexity, we utilize the KD technique, which consists of two components: the teacher model, T-KAN, and the student model, S-CNN. For the teacher model, T-KAN, to capture richer feature representations while reducing the space complexity, we employ Kolmogorov-Arnold Networks (KAN) by replacing all MLP layers with learnable B-spline functions. This allows the model to maintain high expressiveness with fewer parameters. For the student model, S-CNN, to maintain a lightweight structure with fewer parameters, we utilize a simplified Convolutional Neural Network (CNN) architecture. This ensures that the model remains efficient and suitable for deployment on resource-constrained devices.

- To address non-stationary time variations and event distribution shifts, we decompose the signal into high and low frequency components. This enables more effective identification and analysis of periodic and trend changes in transportation modes. By separating these components, we reduce the impact of event distribution shifts on trend data, thereby reducing the effects of non-stationary time variations.
- To enhance the model’s non-linear fitting ability and interpretability, we use KAN. Based on the Kolmogorov-Arnold representation theorem, KAN represents multivariate continuous functions as combinations of univariate functions and additions [18]. Unlike traditional neural networks, KAN has learnable activation functions on edges, typically splines, which replace weight parameters. This design boosts flexibility, reduces parameters, and improves interpretability.
- To reduce time and space complexity, we use knowledge distillation. This involves training a small student model under the guidance of a large teacher model, transferring the teacher’s knowledge to the student [19]. The student model can learn the behavior and decisions of the teacher model without having the same number of parameters, resulting in model compression and speedup. Through this, we lower the model’s complexity while retaining near-original performance. In TMD, this means that smaller and more efficient models can be used to handle large-scale datasets without significantly compromising accuracy, thus improving the utility and scalability of the model.

## Related work

TMD technology has advanced from GPS-based approaches to multi-sensor integration and machine learning, and now to deep learning. This progression has significantly enhanced recognition accuracy and efficiency while laying the groundwork for intelligent transportation systems (Table 1).

**Table 1:** Summary of previous TMD approaches.

Method category	Strengths	Limitations	Ref.
GPS-based	Global coverage and fairly acccurate	Signal occlusion, energy-intensive	[6–8, 20–23]
Multi-sensor with ml	Improved accuracy over GPS-only	Time-consuming extraction, subjective	[9–12, 24–27]
Multi-sensor with dl	Advanced accuracy	Computationally intensive, overfitting	[13–16, 28–36]

**Note:** ml: machine learning, dl: deep learning

### GPS-based

Initial TMD techniques primarily used GPS data due to its global coverage and precision. Gong *et al*. [6] developed a GIS algorithm for processing GPS trip data, achieving an 82.6% success rate in identifying travel modes in NYC. Li *et al*. [7] combined GPS and GIS data with random forest algorithms for mode identification, while Zheng *et al*. [21] proposed supervised learning methods to identify transportation modes from raw GPS logs. Despite these advances, GPS-based methods face limitations from signal occlusion and high energy consumption.

### Multi-sensor integration with machine learning

The integration of additional sensors like accelerometers and gyroscopes represented a significant advancement in TMD development. Feng *et al*. [24] demonstrated that accelerometer-only methods outperformed GPS-only ones, with the combination of both yielding the highest accuracy. Machine learning algorithms, including random forests and XGBoost [9–11], were employed to process these multi-source data. While this approach enhanced recognition capabilities, it remained limited by manual feature extraction, which is time-consuming and subjective.

### Multi-sensor integration with deep learning

Recent years have seen deep learning significantly advance TMD through automatic feature extraction. CNNs [28–31], RNNs [36], and Transformers [35] have been applied with varying success. Many other deep learning methods have also been applied, for example, Wang [33] introduced T2Trans using temporal convolutional networks, while Asci *et al*. [34] employed LSTM for TMD. Despite these advances, existing deep learning models often struggle with complex temporal analysis and maintaining a balance between model complexity and performance.

Technology has transformed TMD, allowing systems to manage intricate data and achieve higher accuracy in results. However, developing a model that can perform complex temporal analyse, offer high accuracy, and maintain a slim profile is still a difficult task.

## Methodology

### Overview

**Fig 2:**
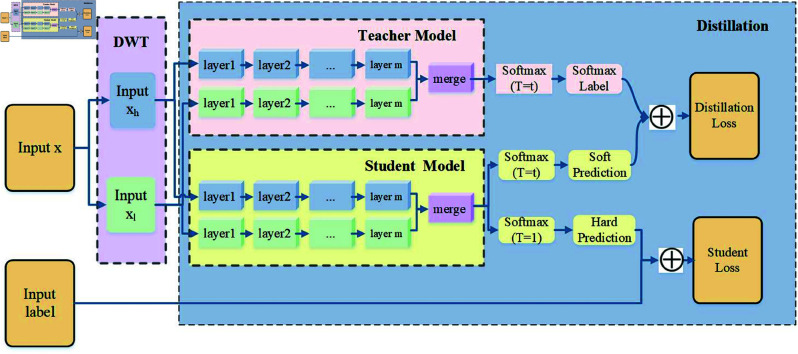
The framework of KDTMD model.

**Fig 3:**
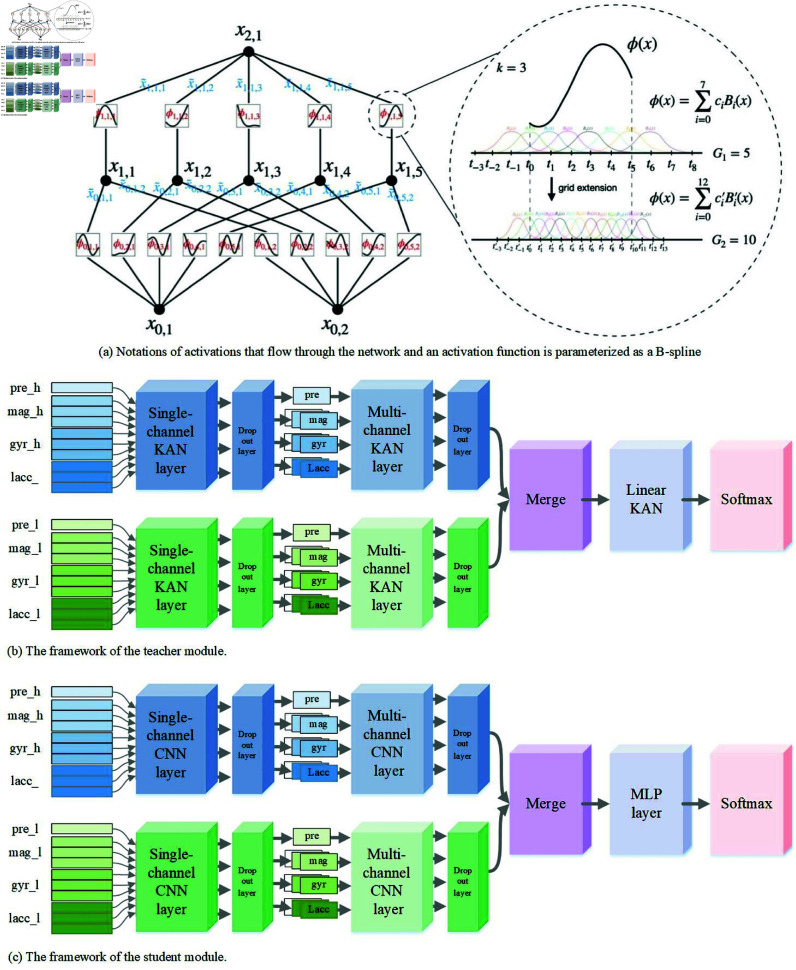
The detailed architecture of the KDTMD model.

In this paper, we propose a lightweight transportation mode detection framework, KDTMD, based on Discrete Wavelet Transform (DWT) and Knowledge Distillation (KD)(We published our proposed KDTMD algorithm at the following website: https://github.com/RuiLi221/KDTMD). The objective is to create a lightweight model with a low number of trainable parameters while maintaining high efficiency. The KDTMD model is designed to be easily deployable on smart wearable devices with limited computing power and storage capacity. As illustrated in Fig 2, the KDTMD model primarily consists of two parts: (i) DWT for capturing non-stationary temporal changes and spatial dynamics, and (ii) KD for reducing time-space complexity and ensuring model lightweighting. The framework includes two main components: (a) a teacher model composed of KAN, and (b) a student model composed of convolutional layers. Specifically, the sensor data $𝒳\in {\mathbb{R}}^{T\times F}$, where *T* denotes the length of the sliding window and *F* represents the number of sensor elements, is initially fed into the DWT. This process disentangles the sensor time series into event representations ${𝒳}_{h}\in {\mathbb{R}}^{T\times F}$ and trend representations ${𝒳}_{l}\in {\mathbb{R}}^{T\times F}$. These two disentangled signals, ${𝒳}_{h}$ and ${𝒳}_{l}$, are then simultaneously fed into both the teacher and student models in the KD module. The goal of this module is to transfer the generalization ability of a complex teacher model to a smaller student model. Our proposed teacher model, based on the KAN, effectively captures the complex nonlinear relationships and dynamic changes in transportation modes, thereby enhancing recognition capabilities. When the high-frequency signal ${𝒳}_{h}$ and the low-frequency signal ${𝒳}_{l}$, along with their corresponding spatiotemporal features, are input into the KAN-based teacher model, this model uses multilayer linear KAN with weight parameters in the form of spline functions to efficiently extract the relevant spatiotemporal features ${𝒳}_{h}^{T}\in {\mathbb{R}}^{T^{T}\times F^{T}}$ and ${𝒳}_{l}^{T}\in {\mathbb{R}}^{T^{T}\times F^{T}}$. In the student model, we use a Convolutional Neural Network (CNN) to process the high-frequency signal ${𝒳}_{h}$ and the low-frequency signal ${𝒳}_{l}$. This CNN efficiently extracts the corresponding spatiotemporal features ${𝒳}_{h}^{S}\in {\mathbb{R}}^{T^{S}\times F^{T}}$ and ${𝒳}_{l}^{S}\in {\mathbb{R}}^{T^{S}\times F^{S}}$ through its local receptive fields and weight-sharing mechanisms. This approach not only reduces the number of parameters but also enhances the model’s ability to learn spatiotemporal hierarchies. In summary, the KDTMD framework leverages the strengths of DWT and KD to create a lightweight, efficient model suitable for deployment on devices with limited resources, while maintaining high accuracy in TMD (Fig 3).

### DWT

The Bayesian structural time series model [37] suggests that traffic time series consist of a stable long-term trend and volatile events, which are independent of each other. This independence, based on the independent mechanisms assumption [38], implies that when one component of the traffic time series changes due to distribution shifts, the other can remain constant. Building on this concept, we enhance model adaptability to non-stationary temporal changes by separating traffic time series into distinct components. By integrating Discrete Wavelet Transform (DWT) into our framework, we effectively disentangle traffic time series into more manageable elements.

**Fig 4:**
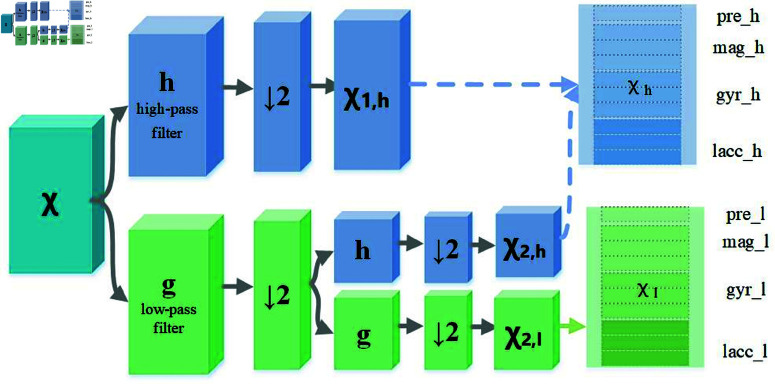
The framework of DWT module in the KDTMD model.

As in a two-level DWT (as shown in Fig 4), the input signal $𝒳\in {\mathbb{R}}^{T}$ is decomposed into a low-frequency component ${𝒳}_{2,l}\in {\mathbb{R}}^{\frac{T}{4}}$ that captures the trend, and two high-frequency components ${𝒳}_{2,h}\in {\mathbb{R}}^{\frac{T}{4}}$ and ${𝒳}_{1,h}\in {\mathbb{R}}^{\frac{T}{2}}$ that capture events, as shown in Fig 4. Here, **g** and **h** denote the low-pass and high-pass filters of the wavelet. For a traffic time series $𝒳\in {\mathbb{R}}^{T_{1}\times N\times C}$, multi-level wavelet transforms with these filters can extract a smooth low-frequency trend and multiple high-frequency event components. The DWT on input traffic data 𝒳 can be formulated as follows (⋆ denotes convolution, and ↓2 indicates output downsampling by 2.):

$${\overset{―}{𝒳}}_{2,l}={\left(𝐠⋆(𝐠⋆𝒳{)}_{\left(↓2\right)}\right)}_{\left(↓2\right)},$$ (1)

$${\overset{―}{𝒳}}_{2,h}={\left(𝐡⋆(𝐠⋆𝒳{)}_{\left(↓2\right)}\right)}_{\left(↓2\right)},$$ (2)

$${\overset{―}{𝒳}}_{1,h}=(𝐡⋆𝒳{)}_{\left(↓2\right)},$$ (3)

After DWT, the low and high frequency components have reduced time steps due to downsampling. To match the input length and return frequency data to the time domain, we apply upsampling and IDWT with inverse filters ${𝐠}^{T}$ and ${𝐡}^{T}$. Also, we sum all inverse high-frequency components as events to retain non-stationary information without adding many channels.

We adopted the fully connected module after IDWT. This design can not only avoid information loss caused by discarding high-frequency components, but also prevent the increase in computational load caused by parallel processing of all high-frequency components. This module converts trends and events into high-dimensional representations ${𝒳}_{l},{𝒳}_{h}\in {\mathbb{R}}^{T_{1}\times N\times d}$, thereby enhancing the expressiveness of the subsequent spatio-temporal network. The formulations for the IDWT and fully-connected module are expressed as Eq 4 (${\text{W}}^{\text{g}}$, ${\text{W}}^{\text{h}}$, ${\text{b}}^{\text{g}}$, ${\text{b}}^{\text{h}}$ are learnable parameters):

$${𝒳}_{l}=W^{g}{𝐠}^{T}⋆{\left({𝐠}^{T}⋆{\left({\overset{―}{𝒳}}_{2,l}\right)}_{↑2}\right)}_{↑2}+b^{g},$$ (4)

$${𝒳}_{h}=W^{h}({𝐠}^{T}⋆{\left({𝐡}^{T}⋆{\left({\overset{―}{𝒳}}_{2,h}\right)}_{↑2}\right)}_{↑2}+\;{𝐡}^{T}⋆{\left({\overset{―}{𝒳}}_{1,h}\right)}_{↑2})+b^{h},$$ (5)

Following the disentangling flow layer, we extract the separated trend and event representations from the traffic data, thereby reducing the impact of non-stationary time variations. Through experimentation, we select the most suitable wavelet from widely used ones to decompose the sensor time series effectively. For details on the selection process, see the “Effect of different hyperparameters” section in Experiments, which explores various wavelet basis functions in DWT.

### Knowledge distillation

In the knowledge distillation module, we design the teacher model with adequate trainable parameters and the lightweight student model with limited parameters. On one hand, the student model can learn as much knowledge as possible from the teacher model, thereby imitating the teacher model’s predictive capabilities. On the other hand, the student model can refer to the input label values for learning, correcting any potential erroneous knowledge it may have learned [19].

#### KAN.

KAN leverages the Kolmogorov-Arnold theorem, which provides a method for expressing continuous multivariable functions as a sum of single-variable functions [18]. As shown in Eq 6, for a smooth function $f:[0,1{]}^{n}\to \mathbb{R}$:

$$f(𝐱)=f\left(x_{1},\cdots ,x_{n}\right)=\sum_{q=1}^{2n+1}\Phi_{q}\left(\sum_{p=1}^{n}\phi_{q,p}\left(x_{p}\right)\right)$$ (6)

where $\phi_{q,p}:[0,1]\to \mathbb{R}$ and $\Phi_{q}:\mathbb{R}\to \mathbb{R}$. Unlike MLPs, which use fixed activation functions on their nodes, KAN feature learnable activation functions on their edges. In KAN, there are no linear weights, every weight parameter is replaced by a univariate function parametrized as a spline, as shown in Fig 3a. The functions that are computed on the edges are represented as B-splines with the spline parameters being the learnable parameters of the network. Compared to MLPs, KAN provide a more nuanced way of capturing complex patterns in data, and are better at modeling complex real valued functions than MLP, thus better mimicking the way information is encoded in sensors. This design enhances the model’s ability to express intricate features, making it more flexible and adaptable to diverse data distributions. Additionally, KAN improve interpretability by allowing intuitive visualization of the learned functions, making the model’s decision-making process more transparent. For TMD problems, these univariate functions can automatically adjust their coefficients based on different characteristics of input data, such as gyroscope, acceleration, and magnetic inputs. This adaptability allows the model to handle various data patterns, providing more nuanced feature expressions for training and enhancing the model’s accuracy and interpretability.

#### Teacher model.

As shown in Fig 3b, we adopt an efficient teacher model, T-KAN, which consists of multiple layers of linear KAN. Specifically, our proposed T-KAN includes modules for processing high-frequency signals, low-frequency signals, fusion, and classification. The high-frequency signal module is composed of a single-channel KAN layer, a multi-channel KAN layer, and a dropout layer. Similarly, the low-frequency signal module has a similar structure to the high-frequency signal module. When signals ${𝒳}_{h}$ and ${𝒳}_{l}$ are fed into the high and low-frequency signal processing modules respectively, they first pass through the single-channel KAN layer to extract the univariate spatiotemporal features of high-frequency signals: ${𝒳}_{h}^{A^{x}}$, ${𝒳}_{h}^{A^{y}}$, and so on, and the univariate spatiotemporal features of low-frequency signals: ${𝒳}_{l}^{A^{x}}$, ${𝒳}_{l}^{A^{y}}$, and so on (as shown in Table 2). In the model, the number of units for all KAN layers is set to 8, the grid size is set to 10, and the pooling size of the pooling layer is set to 0.2. The time dimension of the input *T* = 500, and the feature dimension *D* = 1, $T^{\prime }=500$ and $D^{\prime }=8$.

**Table 2:** Variable names and descriptions for univariate and integrated spatiotemporal features.

Name	Description	Name	Description
$T^{\prime }$	Temporal dim for each axis of sensor	$T^{\prime \prime }$	Temporal dim for each sensor
$D^{\prime }$	Feature dim for each axis of sensor	$D^{\prime \prime }$	Feature dim for each sensor
${𝒳}_{h}^{A^{x}},{𝒳}_{h}^{A^{y}},{𝒳}_{h}^{A^{z}}\in {\mathbb{R}}^{T^{\prime }\times D^{\prime }}$	High-frequency of lacc of x, y, z	${𝒳}_{h}^{A^{\prime }}\in {\mathbb{R}}^{T^{\prime \prime }\times D^{\prime \prime }}$	High-frequency of lacc
${𝒳}_{h}^{G^{x}},{𝒳}_{h}^{G^{y}},{𝒳}_{h}^{G^{z}}\in {\mathbb{R}}^{T^{\prime }\times D^{\prime }}$	High-frequency of gyr of x, y, z	${𝒳}_{h}^{G^{\prime }}\in {\mathbb{R}}^{T^{\prime \prime }\times D^{\prime \prime }}$	High-frequency of gyr
${𝒳}_{h}^{M^{x}},{𝒳}_{h}^{M^{y}},{𝒳}_{h}^{M^{z}}\in {\mathbb{R}}^{T^{\prime }\times D^{\prime }}$	High-frequency of mag of x, y, z	${𝒳}_{h}^{M^{\prime }}\in {\mathbb{R}}^{T^{\prime \prime }\times D^{\prime \prime }}$	High-frequency of mag
${𝒳}_{h}^{P}\in {\mathbb{R}}^{T^{\prime }\times D^{\prime }}$	High-frequency of bar	${𝒳}_{h}^{P^{\prime }}\in {\mathbb{R}}^{T^{\prime \prime }\times D^{\prime \prime }}$	High-frequency of bar
${𝒳}_{l}^{A^{x}},{𝒳}_{l}^{A^{y}},{𝒳}_{l}^{A^{z}}\in {\mathbb{R}}^{T^{\prime }\times D^{\prime }}$	Low-frequency of lacc of x, y, z	${𝒳}_{l}^{A^{\prime }}\in {\mathbb{R}}^{T^{\prime \prime }\times D^{\prime \prime }}$	Low-frequency of lacc
${𝒳}_{l}^{G^{x}},{𝒳}_{l}^{G^{y}},{𝒳}_{l}^{G^{z}}\in {\mathbb{R}}^{T^{\prime }\times D^{\prime }}$	Low-frequency of gyr of x, y, z	${𝒳}_{l}^{G^{\prime }}\in {\mathbb{R}}^{T^{\prime \prime }\times D^{\prime \prime }}$	Low-frequency of gyr
${𝒳}_{l}^{M^{x}},{𝒳}_{l}^{M^{y}},{𝒳}_{l}^{M^{z}}\in {\mathbb{R}}^{T^{\prime }\times D^{\prime }}$	Low-frequency of mag of x, y, z	${𝒳}_{l}^{M^{\prime }}\in {\mathbb{R}}^{T^{\prime \prime }\times D^{\prime \prime }}$	Low-frequency of mag
${𝒳}_{l}^{P}\in {\mathbb{R}}^{T^{\prime }\times D^{\prime }}$	Low-frequency of bar	${𝒳}_{l}^{P^{\prime }}\in {\mathbb{R}}^{T^{\prime \prime }\times D^{\prime \prime }}$	Low-frequency of bar

**Note:** dim: dimension, lacc: linear acceleration, gry: gyroscope, mag: magnetometer, bar: barometer

The univariate spatiotemporal features for each sensor are then passed through the Multi-channel KAN layer for further feature learning, producing the integrate spatiotemporal features of high-frequency: ${𝒳}_{h}^{A^{\prime }}$, ${𝒳}_{h}^{G^{\prime }}$, ${𝒳}_{h}^{M^{\prime }}$, ${𝒳}_{h}^{P^{\prime }}$, and the integrate spatiotemporal features of low-frequency: ${𝒳}_{l}^{A^{\prime }}$, ${𝒳}_{l}^{G^{\prime }}$, ${𝒳}_{l}^{M^{\prime }}$, ${𝒳}_{l}^{P^{\prime }}$, (show in Table 2) where $T^{\prime \prime }=500$, $D^{\prime \prime }=8$.

Then all the integrated spatiotemporal features of high-frequency and low-frequency above are fused together in the merge layer to form feature ${𝒳}^{m}\in {\mathbb{R}}^{T^{m}\times D^{m}}$ where *T*^*m*^ = 500 and *D*^*m*^ = 32. these features are then transformed by the linear KAN layer into the final feature vector ${𝒳}^{f}\in {\mathbb{R}}^{D^{f}}$ where *D*^*f*^ = 8. Finally, the softmax layer is used to classify the corresponding transportation mode.

#### Student model.

In this section, we introduce a streamlined student model called S-CNN (show in Fig 3c) and enhance its training efficiency through the application of knowledge distillation. Similar to the teacher model, the student model also includes modules for processing high-frequency signals, low-frequency signals, fusion, and classification, However, compared to the teacher model, the student model has a simpler structure and fewer parameters, The filters of all CNN is set to 3, the kernel size is set to 3 and the units of MLP layer is set to 8. The high-frequency signal module of the student model is composed of a Single-channel CNN layer and a Multi-channel CNN layer, and similarly, the low-frequency signal module of the student model adopts the same structure. To facilitate the subsequent knowledge distillation task, the student model and the teacher model maintain the same input, that is, the student model is also fed with event representations ${𝒳}_{h}\in {\mathbb{R}}^{T\times F}$ and trend representations ${𝒳}_{l}\in {\mathbb{R}}^{T\times F}$, which are then processed through the Single-channel CNN layer and Multi-channel CNN layer for feature extraction to form the integrate spatiotemporal features of high-frequency: ${𝒳}_{h}^{{SA}^{\prime }}$, ${𝒳}_{h}^{{SG}^{\prime }}$, ${𝒳}_{h}^{{SM}^{\prime }}$, ${𝒳}_{h}^{{SP}^{\prime }}$, and the integrate spatiotemporal features of low-frequency: ${𝒳}_{l}^{{SA}^{\prime }}$, ${𝒳}_{l}^{{SG}^{\prime }}$, ${𝒳}_{l}^{{SM}^{\prime }}$, ${𝒳}_{l}^{{SP}^{\prime }}$.

Then all the integrated spatiotemporal features of high-frequency and low-frequency above are fused together in the merge layer to form feature ${𝒳}_{s}^{m}\in {\mathbb{R}}^{T_{s^{m}}\times D_{s^{m}}}$ where $T_{s^{m}}=500$, $D_{s^{m}}=12$. these features are then transformed by the MLP layer into the final feature vector ${𝒳}_{s}^{\;f}\in {\mathbb{R}}^{D_{s^{\;f}}}$ where $D_{s^{\;f}}=8$. Finally, the softmax layer is used to classify the corresponding transportation mode.

#### Distillation training.

Within the knowledge distillation framework, we define both the complex teacher model with better generalization ability and the lightweight student model with limited parameters. On one hand, the student model can learn as much knowledge as possible from the teacher model, thereby imitating the teacher model’s predictive capabilities. On the other hand, the student model can refer to the input label values for learning, correcting any potential erroneous knowledge it may have learned. Specifically, we use a “softmax” output layer to generate class probabilities, which is a common practice in neural networks. Different from the ordinary softmax, we introduced the concept of temperature to better express the latent importance of each predicted value. The specific implementation is as follows: First, we obtain the corresponding values from the output of the last fully connected layer of both the teacher and the student. Then, we take the logits to get $z_{j}^{t}$ and $z_{j}^{s}$. After that, we define the soft targets $p\left(z_{i}^{t},T\right)$ and $p\left(z_{i}^{s},T\right)$ (show in Eqs 7, 8) which represents the probability that the sample belongs to the i-th category for the teacher and student, respectively.

$$p\left(z_{i}^{t},T\right)=\frac{\exp\left(z_{i}^{t}/T\right)}{\sum_{j}\exp\left(z_{j}^{t}/T\right)}$$ (7)

$$p\left(z_{i}^{s},T\right)=\frac{\exp\left(z_{i}^{s}/T\right)}{\sum_{j}\exp\left(z_{j}^{s}/T\right)}$$ (8)

The final loss of the entire model is composed of two parts. One part is the distillation loss ${ℒ}_{D}$, which represents the loss of knowledge transferred from the teacher to the student at temperature T. ${ℒ}_{D}$ is defined as the cross-entropy loss determined by $p\left({𝐳}_{i}^{t},T\right)$ and $p\left({𝐳}_{i}^{s},T\right)$ (as shown in Eq 9). The other part is the student loss ${ℒ}_{S}$, which represents the loss of knowledge learned by the student from the input labels at temperature 1 (as shown in Eq 10). The final loss of the model is the sum of these two parts (as shown in Eq 11), where α is the distillation factor.

$${ℒ}_{D}\left(p\left({𝐳}_{i}^{t},T\right),p\left({𝐳}_{i}^{s},T\right)\right)=-\sum^{N}p\left({𝐳}_{i}^{t},T\right)\log\left(p\left({𝐳}_{i}^{s},T\right)\right)$$ (9)

$${ℒ}_{s}\left(y_{i}^{s},p\left({𝐳}_{i}^{s},T\right)\right)=-\sum_{i}^{N}y_{i}^{s}\log\left(p\left({𝐳}_{i}^{s},T\right)\right)$$ (10)

$$ℒ=(1-\alpha ){ℒ}_{D}+\alpha {ℒ}_{s}$$ (11)

### The output

Finally, based on the final features ${𝒳}_{s}^{\;f}$ and the final loss of the model ℒ, we classified the transportation mode and obtained the final output ${𝒳}^{O}\in {\mathbb{R}}^{8}$, formulated as:

$${𝒳}^{O}=softmax({𝒳}^{F})$$ (12)

We investigate the effectiveness of our KDTMD with the goal to answer the following research questions:

**RQ1:** Does KDTMD outperform other baselines?

**RQ2:** How do hyper-parameters (e.g., temperature, alpha) affect KDTMD?

**RQ3:** How do different components in KDTMD affect model performance?

**RQ4:** How does KDTMD perform in terms of computational and resource efficiency?

## Experiments

### Datasets

We assessed the effectiveness of the KDTMD (Knowledge Distillation for Transportation Mode Detection) algorithm using the SHL [39] and HTC [40] real-world datasets. To optimize the model for edge devices, we only utilized low-consumption sensors, including the gyroscope (gyr: x, y, z in ${\text{rad s}}^{-1}$), linear accelerometer (lacc: x, y, z in ${\text{m s}}^{-2}$), and magnetometer (mag: x, y, z in μT). For the SHL dataset, we also included barometric pressure (pre: in hPa). The data preprocessing steps included normalization and segmentation. Subsequently, the data were split into training, validation, and test sets at a ratio of 70%, 20%, and 10%, respectively.

#### SHL dataset.

The Sussex-Huawei Locomotion-Transportation (SHL) dataset, collected over 7 months in the UK and totaling approximately 753 hours, captures various real-life transportation modes such as standing, walking, running, cycling, driving, taking the bus, train, or subway. It includes 3-axis accelerometers, gyroscopes, linear accelerometers, magnetometers, orientation sensors, and a barometer, all sampled at 100 Hz. We utilized a subset of this dataset to evaluate our algorithm, totaling approximately 390 hours. The durations of different transportation modes in SHL dataset are shown in the Table 3.

**Table 3:** Durations of different transportation modes in SHL dataset.

	Original	Selected
Still	127h	65h
Walk	127h	66h
Run	21h	12h
Bike	79h	42h
Car	88h	46h
Bus	107h	56h
Train	115h	60h
Subway	89h	43h
Total	753h	390h

#### HTC dataset.

The HTC dataset, collected from 150 HTC smartphone users, contains 100GB of data across 8,311 hours of various activities recorded at 100Hz from accelerometers, gyroscopes, and magnetometers. The dataset was gathered through two primary avenues: a university program involving 150 participating students and a group of 74 employees and interns. While it initially included activities like motorcycle riding and high-speed rail travel, we removed these to align with the SHL dataset. This resulted in a substantial and consistent dataset for assessing the scalability of our KDTMD model. The durations of different transportation modes in HTC dataset are shown in Table 4

**Table 4:** Durations of different transportation modes in HTC dataset.

	In Prog	Univ Prog	Selected
Still	107h	1750h	1750h
Walk	107h	1263h	1263h
Run	61h	88h	88h
Bike	78h	61h	61h
Motorcycle	134h	1683h	0h
Car	209h	558h	558h
Bus	69h	1248h	1248h
Metro	95h	289h	289h
Train	67h	267h	267h
HSR	91h	72h	0h
Total	1032h	7279h	5524h

#### Normalization.

To deal with the inconsistency of dimension and numerical range between data from different sensors, we performed Z-Score normalization for the individual components of the sensors. This step helps to calibrate the data so that it has a uniform scale benchmark, which can be expressed by the following formula (formula 13):

$$x^{\prime }=\frac{x-\mu }{\sigma }$$ (13)

where μ represents the mean value of each component, and σ is the standard deviation of the corresponding component.

#### Segmentation.

To enhance accuracy while maintaining manageable computational complexity and processing time, we employed a fixed-length sliding window technique to segment continuous sensor time series into shorter, more manageable pieces for feature analysis. Specifically, the SHL dataset used a window length of 500, whereas the HTC dataset employed a window length of 450. These window lengths were chosen to provide an appropriate scale of data for feature learning in both datasets.

### Baselines

- *RF:* Random Forest (RF) enhances the ensemble’s prediction accuracy through the combination of multiple decision trees [41].
- *MLP:* The Multilayer Perceptron (MLP) extracts and learns features from the data through multiple fully connected layers of neurons [16].
- *CNN:* Convolutional Neural Networks (CNNs) capture spatial features in data through convolutional and pooling layers [42].
- *LSTM:* Long Short-Term Memory (LSTM) maintain the temporal dependencies in sequence data through their gated architecture, effectively capturing long-term patterns and relationships [43].
- *T2Trans:* T2Trans, founded on Temporal Convolutional Networks (TCNs), utilizes the properties of temporal convolution to bolster the precision of TMD [33].
- *CL:* CL-TRANSMODE(CL) consists of three layers: data preprocessing, a CNN for feature extraction, and an LSTM network with dropout for enhanced learning [13].
- *MSRLSTM:* MSRLSTM integrates residual and LSTM layers to extract features from sensor data, leveraging a residual unit to accelerate learning and an attention model to enhance recognition accuracy [16].
- *MSCPT:* Multi-sensor cross-place transportation mode recognition algorithm (MSCPT) comprises three main components: a Multi-Sensor Neural Network model, a variant of the bootstrap ensemble learning method, and data augmentation strategies [42].
- *MLI:* Multimodal Learning Integrator (MLI)The Learning Integrator (MLI) consists of a four-layer hierarchical neural network and Random Forest (RF) classifiers [44].

### Metrics

We assessed model performance using four key metrics: accuracy, precision (14), recall (15), and F1 score (16). Accuracy reflects the model’s overall ability to correctly classify instances; precision focuses on the accuracy of the model’s positive predictions; recall measures the model’s ability to identify all positive instances; and the F1 score is the harmonic mean of precision and recall, providing a comprehensive reflection of the model’s classification performance. (16):

$$\text{Precision}=\frac{1}{N_{m}}\sum_{i=1}^{c}P_{i}$$ (14)

$$\text{Recall}=\frac{1}{N_{m}}\sum_{i=1}^{c}R_{i}$$ (15)

$$\text{F1-Score}=\frac{2\times \text{Precision}\times \text{ Recall}}{\text{Precision}+\text{ Recall}}$$ (16)

where *N*_*m*_ = 8 represents the number of transportation modes.

### Experimental Settings

We utilized the wavelet transform functions provided by the PyWavelets (pywt) library to perform DWT. Within the Keras deep learning framework, we defined a Distiller model and implemented the calculation of the total distillation loss, which includes both the distillation loss and the student loss. We employed the Adam optimizer with an initial learning rate of 0.001. The model was trained for 150 epochs with a batch size of 256, and the data input order was shuffled during training to mitigate overfitting. Additionally, we provided the configuration details and relevant software versions (shown in Table 5).

**Table 5:** Configuration and related software version.

Name	Detail
GPU	RTX 2080 Ti(11GB) * 1
CPU	Xeon Platinum 8255C 2.5GHz
Memory	43GB
CUDA	12.4
Operating System	Ubuntu 20.04.4 LTS
Python Version	Python 3.8.10
Development Framework	Keras, sklearn

### Results

#### Comparison with different baselines (RQ1).

**Fig 5:**
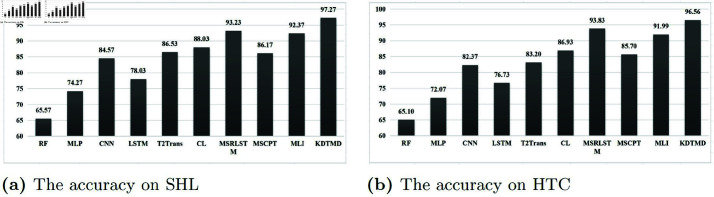
The accuracy of different algorithms for TMD on SHL and HTC datasets.

**Fig 6:**
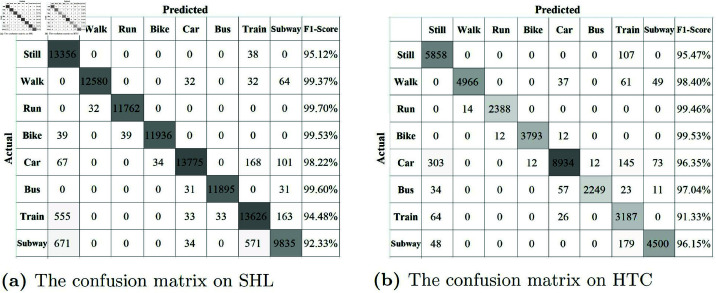
The confusion matrix of KDTMD model on SHL and HTC datasets.

As demonstrated in Figs 5, 6, Tables 6, 7, and 8, the results show that deep learning algorithms such as MLP, CNN, and LSTM generally outperform traditional machine learning methods. The accuracy of single deep learning models typically ranges from 75% to 85%, while ensemble methods like CL-TRANSMODE, MSRLSTM, and MLI achieve accuracy rates mostly above 85%, surpassing individual deep learning models. This indicates that deep learning algorithms possess superior feature extraction capabilities. Hybrid frameworks that combine multiple networks can leverage the strengths of each approach, effectively capturing the spatiotemporal characteristics of sensor data and resulting in higher predictive accuracy for TMD. Among these, the MSRLSTM algorithm achieved the highest accuracy compared to other deep learning methods. This improvement suggests that MSRLSTM enhances LSTM by incorporating residual units and attention mechanisms, leading to stronger feature representation and significantly better predictive accuracy than standard LSTM (78.03% on SHL and 76.73% on HTC).

**Table 6:** F1-Score of different algorithms for TMD on SHL dataset.

	RF	MLP	CNN	LSTM	T2Trans	CL	MSRLSTM	MSCPT	MLI	KDTMD
Still	66.24%	75.60%	86.25%	81.88%	85.52%	85.07%	89.26%	84.01%	91.11%	95.12%
Walk	86.36%	93.32%	96.81%	93.44%	96.79%	98.62%	99.37%	97.19%	99.49%	99.37%
Run	97.16%	98.32%	99.03%	98.88%	98.90%	99.32%	99.86%	99.23%	99.59%	99.70%
Bike	84.82%	88.61%	95.72%	91.12%	96.87%	98.05%	99.51%	96.75%	99.84%	99.53%
Car	64.21%	74.10%	86.38%	78.88%	85.12%	89.14%	90.93%	86.72%	92.86%	98.22%
Bus	30.37%	60.45%	84.57%	66.93%	77.91%	82.51%	90.27%	79.54%	88.89%	99.60%
Train	44.44%	53.95%	68.19%	60.56%	79.14%	76.69%	88.89%	76.29%	85.18%	94.48%
Subway	38.64%	53.01%	60.73%	55.25%	72.58%	75.65%	88.85%	72.73%	82.24%	92.33%

**Table 7:** F1-Score of different algorithms for TMD on HTC dataset.

	RF	MLP	CNN	LSTM	T2Trans	CL	MSRLSTM	MSCPT	MLI	KDTMD
Still	46.71%	70.12%	84.29%	75.29%	82.51%	78.79%	94.85%	87.36%	90.21%	95.47%
Walk	89.65%	91.14%	95.39%	91.82%	96.46%	98.48%	99.24%	96.62%	98.24%	98.40%
Run	97.54%	98.74%	99.03%	99.03%	98.88%	99.86%	99.86%	99.17%	99.49%	99.46%
Bike	86.28%	90.63%	95.25%	91.65%	94.72%	98.70%	99.19%	95.58%	97.88%	99.53%
Car	57.54%	71.26%	85.75%	75.56%	86.69%	90.48%	94.21%	89.54%	91.43%	96.35%
Bus	41.64%	53.59%	79.82%	63.81%	80.00%	88.68%	89.77%	82.93%	87.70%	97.04%
Train	37.34%	53.51%	63.46%	58.31%	67.29%	72.46%	89.21%	72.94%	87.45%	91.33%
Subway	29.15%	37.13%	59.87%	58.39%	59.76%	68.00%	84.04%	61.63%	85.32%	96.15%

**Table 8:** The precision, recall, and F1-Scores of the KDTMD model on the SHL dataset.

	Precision	Recall	F1-Score
Still	90.93%	99.72%	95.12%
Walk	99.75%	98.99%	99.37%
Run	99.67%	99.73%	99.70%
Bike	99.72%	99.35%	99.53%
Car	99.07%	97.38%	98.22%
Bus	99.72%	99.48%	99.60%
Train	94.40%	94.56%	94.48%
Subway	96.48%	88.52%	92.33%

The proposed KDTMD model achieved an accuracy of 97.27% on the SHL dataset and 96.56% on the HTC dataset, significantly outperforming the MSRLSTM algorithm and other baseline results. This demonstrates the critical role of the introduced modules in enhancing TMD. Specifically, the DWT module decomposes traffic time series into a low-frequency trend component and a high-frequency event component, mitigating the effects of non-stationary variations. Within the knowledge distillation framework, our teacher model, T-KAN, employs linear KAN layers with learnable B-spline functions to efficiently capture rich features, enhancing the model’s nonlinear capabilities and interpretability. This teacher model guides a lightweight student model, S-CNN, through the distillation process, ensuring rapid and accurate traffic mode detection. Together, these components ensure high precision, swift predictions, and manageable model complexity.

**Fig 7:**
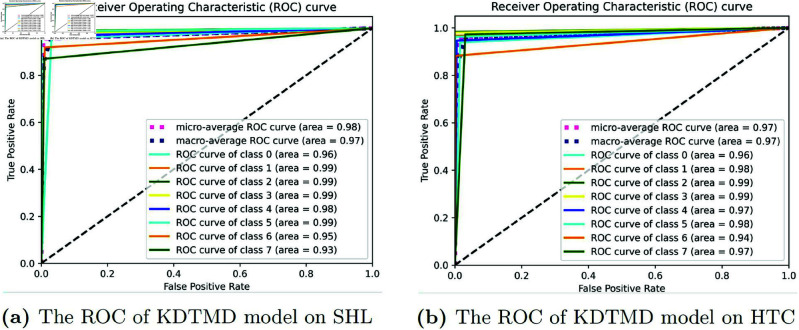
The ROC of KDTMD model on SHL and HTC datasets.

The provided ROC curves illustrate the performance of the KDTMD model on two distinct datasets, SHL and HTC (as shown in Fig 7). For the SHL dataset, the micro-average ROC curve achieves an area under the curve (AUC) of 0.98, indicating excellent overall classification performance. Similarly, the macro-average ROC curve also attains an AUC of 0.97, reflecting consistent performance across all classes. Individual class performances are strong, with most classes achieving AUC values close to 0.99, and even the lower-performing classes (e.g., class 6 and class 7) still demonstrating respectable AUC values of 0.95 and 0.93, respectively. On the HTC dataset, the model maintains a high level of performance, with the micro-average ROC curve achieving an AUC of 0.97. The macro-average ROC curve similarly achieves an AUC of 0.97, indicating robust performance across all classes. Most classes exhibit AUC values above 0.95, with several classes reaching near-perfect AUC values of 0.99. Even the lower-performing classes (e.g., class 0 and class 6) achieve AUC values of 0.96 and 0.94, respectively. Overall, the KDTMD model demonstrates strong classification capabilities on both datasets, with high AUC values across all classes, underscoring its effectiveness in handling multi-class classification tasks.

The Matthews Correlation Coefficient (MCC) values for the KDTMD model on the SHL and HTC datasets are both exceptionally high, at 0.9503 and 0.9449 respectively. These values indicate outstanding classification performance across both datasets. The consistency of these high MCC values across SHL and HTC demonstrates the model’s robustness and reliability in handling multi-class classification tasks, further underscoring its effectiveness in real-world applications.

#### Effect of different hyperparameters (RQ2).

To better understand the impact of each hyperparameter on our KDTMD model, we conducted experiments to tune the hyperparameters of the KDTMD model, we mainly adjusted the following core hyperparameters: (i) different wavelet basis functions in DWT, (ii) different numbers of Units, Grid_size and Spline_order in KAN for teacher model, (iii) different numbers of filters in convolutional layer for student model, (iv) the parameter of temperature in distillation loss, the parameter of alpha in total loss, and the learning rate in knowledge distillation.

**Fig 8:**
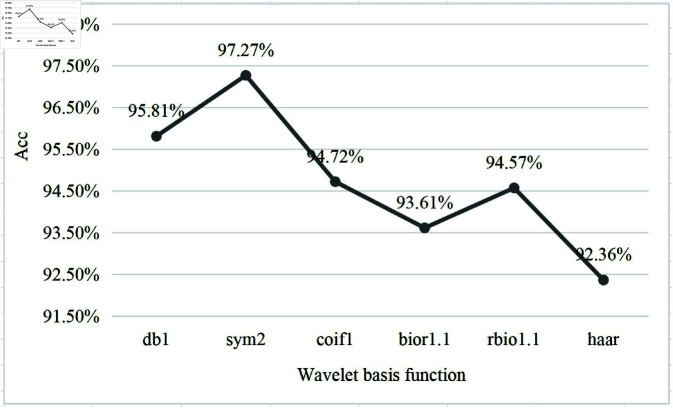
Different wavelet basis functions in DWT.

**Fig 9:**
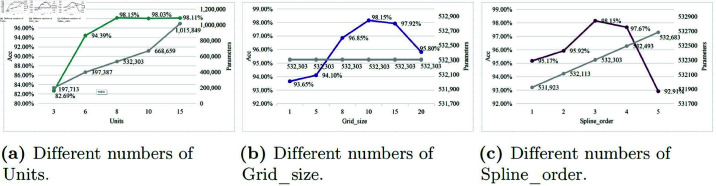
Different numbers of Units, Grid_size and Spline_order in KAN for teacher.

(i) We tested six wavelet basis functions for the wave parameter: db1 (Daubechies 1), sym2 (Symlet 2), coif1 (Coiflet 1), bior1.1 (Biorthogonal 1.1), rbio1.1 (Reverse Biorthogonal 1.1), and haar (Haar).(as shown in Fig 8). In our experiments, the sym2 wavelet basis function demonstrated superior performance in handling non-stationary time variations and event distribution shifts, achieving an accuracy rate of 97.27%. This indicates that the symmetry and higher vanishing moments of sym2 enable it to better capture the trend changes in signals while reducing the impact of event distribution shifts. Therefore, we selected sym2 as the wavelet basis function for our final model.

(ii) We conducted experiments to optimize the teacher KAN model by adjusting three key parameters: Units, Grid_size, and Spline_order (as shown in Fig 9).

- For Units, accuracy improved with increasing units, but the parameter count also rose. The teacher model achieved 98.15% accuracy at units=8. Beyond this, accuracy gains were minimal despite a significant parameter increase (e.g., the parameter count at units=15 was approximately double that of units=8). Thus, units=8 was chosen for balancing accuracy and complexity.
- Regarding grid_size, accuracy showed a consistent upward trend with increasing grid_size, while the parameter count remained stable. The optimal accuracy was achieved at grid_size=10, beyond which no further accuracy enhancement was observed.
- In KAN, the number of B-spline basis functions is determined by grid_size and spline_order, specifically as the sum of grid_size and spline_order. Our experiment fixed grid_size at 10 and varied spline_order from 1 to 5, resulting in B-spline basis functions ranging from 11 to 15. This setup allowed us to observe how changes in the number of B-spline basis functions affect the teacher model’s accuracy. We observed that accuracy generally improved with higher spline_order values, though this came with a modest increase in parameters. However, when spline_order exceeded 3, accuracy began to decline. Therefore, spline_order=3 corresponds to the number of B-spline basis functions being 13, offering the best trade-off between accuracy and parameter efficiency.

(iii) We conducted experiments to evaluate different student models, including variations of MLP, TCN, LSTM, and CNN, with a focus on optimizing CNN through adjustments to key hyperparameters such as filter size, pooling strategy, dropout, and batch normalization. The results are summarized in the Table 9 below:

**Table 9:** Performance comparison of different student models on the SHL dataset.

Name	Precision	Recall	F1-Score	Acc	Parameters	PT(ms)	FLOPs	Description
S-MLP	94.67%	94.35%	94.25%	94.20%	48,329	0.19	2.93e+02 M	variants of MLP as student mode
S-TCN	93.27%	91.28%	91.26%	91.17%	146,531	0.95	4.39e+02 M	variants of TCN as student mode
S-LSTM	85.99%	85.55%	85.72%	85.70%	162,443	3.58	6.88e+02 M	variants of LSTM as student mode
S-CNN1	95.27%	95.29%	95.23%	95.23%	48,803	0.18	2.25e+02 M	CNN(f=3)
S-CNN2	94.88%	94.85%	94.73%	94.73%	32,419	0.17	1.19e+02 M	CNN(f=2), dr(r=0.1)
S-CNN3	97.40%	97.29%	97.29%	97.27%	48,803	0.18	2.25e+02 M	CNN(f=3), dr(r=0.1)
S-CNN4	97.17%	97.18%	97.12%	97.03%	65,307	0.20	3.61e+02 M	CNN(f=4), dr(r=0.1)
S-CNN5	89.96%	89.84%	89.46%	89.57%	38,095	0.16	2.25e+02 M	CNN(f=3), dr(r=0.1), pl(ps=4)
S-CNN6	91.88%	91.52%	91.15%	91.17%	48,985	0.22	2.25e+02 M	CNN(f=3), dr(r=0.1), with bn

**Note:** dr: dropout, r:rate, pl: pooling, ps: pool_size, f: filters, bn: batch normalization, PT: Predicting Time

**Fig 10:**
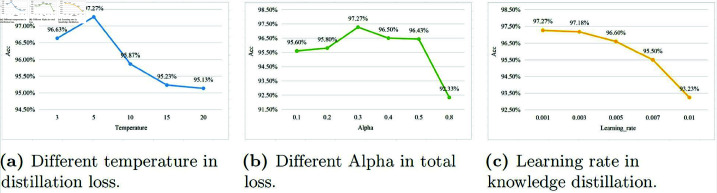
Different hyperparameters in knowledge distillation.

The table presents a comprehensive comparison of various student models in terms of their performance metrics, computational efficiency, and architectural details. Here’s a detailed analysis:

- S-CNN1 (filter=3) demonstrates superior accuracy compared to S-TCN and S-LSTM, with a notably higher ACC of 95.23%. Moreover, its Predicting Time (0.18 ms) is significantly shorter than that of S-TCN (0.95 ms) and S-LSTM (3.58 ms), making it more suitable for TMD systems requiring rapid responses. Compared to S-MLP, which has a similar Predicting Time (0.19 ms), S-CNN achieves a higher ACC (95.23% vs. 94.20%) with fewer FLOPs (2.25e+02 M vs. 2.93e+02 M), indicating better computational efficiency and accuracy.
- S-CNN3, which incorporates dropout (dr(rate=0.1)), shows a improvement in ACC (97.27%) over S-CNN1 (95.23%) with minimal changes in Predicting Time and FLOPs. This suggests that dropout enhances model generalization without compromising efficiency.
- S-CNN2, S-CNN3, and S-CNN4 reveal that increasing filter size (f) boosts model accuracy but also raises parameter count and computational demands. The optimal balance is achieved at f=3, where S-CNN3 attains the highest ACC of 97.27%, indicating that filter size significantly impacts model performance.
- S-CNN5, which applies pooling (pl(ps=4)), exhibits a reduction in parameters but suffers from lower accuracy, implying that pooling may discard crucial information and is thus less effective for this task.
- S-CNN6, equipped with batch normalization (bn), shows a slight increase in parameters and Predicting Time but a decrease in ACC, suggesting that batch normalization may not be as effective as other regularization techniques like dropout in this context.

(iv) We conducted experiments with various hyperparameters in knowledge distillation, including different temperatures in the distillation loss, different Alpha values in the total loss, and learning rates specific to knowledge distillation. The results are presented in Fig 10.

- In knowledge distillation, varying temperature (*T*)(as shown in Eq 17) affects the balance between hard and soft targets, here *z*_*i*_ represents the raw score (logit) computed for each class, while *q*_*i*_ is the probability derived from *z*_*i*_ using the softmax function. At lower *T* values, the model focuses more on hard targets (crisp predictions), while higher *T* values emphasize soft targets (probabilistic distributions). Through extensive testing, we observed that *T* = 5 provides the best trade-off, maximizing accuracy and stability (as shown in Fig 10a). This selection ensures optimal knowledge transfer from the teacher to the student model.
  $$q_{i}=\frac{\exp\left(z_{i}/T\right)}{\sum_{j}\exp\left(z_{j}/T\right)}$$ (17)
- We explored the impact of different alpha values on model performance when calculating the total loss ℒ (as shown in Eq 11), with results presented in Fig 10b. In knowledge distillation, adjusting alpha alters the emphasis between the distillation loss ${ℒ}_{D}$ and student loss ${ℒ}_{s}$. Different alpha values affect the model’s ability to learn from the teacher’s probabilistic guidance. Our experiments revealed that setting alpha to 0.3 optimally balances these two aspects, achieving the highest accuracy and ensuring robust knowledge transfer from the teacher to the student model, leading to superior model performance.
- We explored the impact of different learning rates ranging from 0.001 to 0.01 (as shown in Fig 10c), and found that the accuracy of TMD was highest when the learning rate was set to 0.001.

#### Ablation studies (RQ3).

We conducted ablation studies to assess the impact of removing DWT, replacing KAN with MLP, and modifying the CNN student model architecture. The results are summarized in the Table 10 below:

**Table 10:** Impact of removing dwt, replacing KAN with mlp, and modifying cnn student model architecture.

Name	Precision	Recall	F1-Score	Acc	Parameters	PT(ms)	FLOPs	Description
NoDWT	82.25%	81.43%	81.34%	81.60%	35,059	0.29	3.26e+02 M	KDTMD without DWT
T-MLP	83.35%	83.59%	83.32%	83.23%	48,803	0.18	2.25e+02 M	Teacher Model with MLP instead of KAN
S-CNN3	97.40%	97.29%	97.29%	97.27%	48,803	0.18	2.25e+02 M	Sc CNN(f=3), Mc CNN (f=3), dr(r=0.1)
S-CNN7	96.91%	96.85%	96.86%	96.83%	49,373	0.42	3.63e+02 M	Sc CNN(f1=3, f2=3), Mc CNN (f=3)
S-CNN8	97.21%	97.13%	97.15%	97.10%	50,468	0.58	6.35e+02 M	Sc CNN(f1=3, f2=3), Mc CNN (f3=3, f4=3)

**Note:** Sc CNN: Single-channel CNN layer, Mc CNN: Multi-channel CNN layer, dr: dropout, r:rate, pl: pooling, ps: pool_size, f: filters, bn: batch normalization, PT: Predicting Time

The table presents the results of ablation studies on different components and architectures of the TMD model. Here’s a detailed analysis:

- NoDWT: Removing DWT leads to a significant drop in accuracy (Acc=81.60%) compared to other models, despite having the fewest Parameters (41,059) and lower FLOPs (3.26e+02 M). This indicates that DWT plays a crucial role in enhancing model performance by decomposing sensor time series into low and high frequency events, reducing the impact of non-stationary variations.
- T-MLP: Replacing KAN with MLP in the teacher model results in a significant drop in accuracy to 83.23%. Since the change only involves the teacher model’s architecture, the student model’s computational efficiency remains the same. However, the notable decrease in the student model’s accuracy indicates that This demonstrates that KAN’s B-spline functions enable it to model complex patterns more efficiently with fewer parameters, thus making it more effective than MLP in feature extraction and positively impacting the student model’s performance.
- S-CNN3 achieves an accuracy of 97.27%, while S-CNN7 and S-CNN8 reveal that increasing the layers in the Single-channel CNN layer and Multi-channel CNN layer raises Parameters and FLOPs, yet reduces accuracy to 96.83% and 97.10%, respectively. This indicates potential overfitting and underscores the importance of architectural optimization to prevent performance degradation.

#### Computational and resource efficiency (RQ4).

In order to deeply analyze the computational complexity of the algorithm, we compared the training time, number of parameters, predicting time, and memory usage of various algorithms. The training time for each algorithm was calculated over one hundred epochs, measured in seconds. As shown in Table 11, the KDTMD model has the shortest training time at 19,532 seconds, while MSRLSTM has the longest at 89,753 seconds. In terms of parameters, KDTMD has the fewest parameters (48,803), which is roughly 10% of the smallest baseline parameters. For predicting time, measured in milliseconds, KDTMD is the fastest with 0.18 ms, followed by MLP at 0.19 ms, whereas LSTM is the slowest at 2.96 ms. Regarding memory usage, KDTMD uses the least memory at 500 M (which is the distilled student model and requires further quantization for mobile deployment), approximately 7.7% of that used by MSRLSTM, which consumes the most memory at 6487 M. This indicates that the KDTMD model achieves high accuracy while substantially reducing parameter size, and demonstrates superior performance in training efficiency, prediction speed, and memory usage, making it highly suitable for lightweight deployment.

**Table 11:** Computational and resource efficiency of different algorithms.

Name	Training time(s)	Parameters	Predicting time(ms)	Memory(M)
MLP	52,638	489,613	0.19	792
CNN	45,346	746,901	0.27	929
LSTM	56,728	962,109	2.96	2173
T2Trans	49,867	893,765	0.83	1997
CL	50,471	838,514	0.95	1982
MSRLSTM	89,753	1,258,248	0.67	6487
MSCPT	64,658	820,943	0.51	1836
MLI	69,154	979,087	0.46	2279
KDTMD	19,532	48,803	0.18	500

## Conclusion

This paper focuses on fine-grained TMD. We propose a novel KDTMD model including DWT and KD modules. By combining DWT with KD, our model addresses non-stationary temporal dynamics, nonlinearity, and space complexity while enhancing performance and efficiency. The DWT module decomposes sensor time series into a trend-indicating low-frequency component and an event-indicating high-frequency component, minimizing the impact of event distribution shifts and reducing the effects of non-stationary variations. In our knowledge distillation setup, we utilize an efficient teacher model, T-KAN, which is based on linear KAN layers with learnable B-spline functions for enhanced feature representation and model interpretability. The student model, S-CNN, is trained by T-KAN, accelerating its learning process. Experimental results demonstrate that the KDTMD model achieved high accuracy rates of 97.27% and 96.56% on the SHL and HTC datasets, surpassing other methods. Notably, The KDTMD model achieves high accuracy with only about 10% of the parameters of the smallest baseline model. Furthermore, it also excels in training efficiency, prediction speed, and memory usage, making it ideal for lightweight deployment.

While the model demonstrates strong performance under ideal conditions, maintaining optimal accuracy in high-noise environments poses a challenge. Additionally, adapting the model to accommodate emerging transportation modes remains an area requiring further research and development. Future research will investigate self-supervised learning techniques to enhance the model’s ability to learn from unlabeled data. Additionally, future research will devote attention to refining the model’s inference mechanisms to ensure efficient real-time operation on edge devices. We look forward to applying the KDTMD model to a variety of mobile intelligent services, such as traffic prediction and logistics route optimization, with the aim of enhancing service quality and optimizing user experience.
